# Significant improvement of survival by T2* CMR in thalassemia major

**DOI:** 10.1186/1532-429X-18-S1-P137

**Published:** 2016-01-27

**Authors:** Antonella Meloni, Caterina Borgna-Pignatti, Giovanni Carlo Del Vecchio, Maria Antonietta Romeo, Maria Rita Gamberini, Federico Bonetti, Maria Giovanna Neri, Elisabetta Chiodi, Vincenzo Positano, Alessia Pepe

**Affiliations:** 1 CMR Unit, Fondazione G. Monasterio CNR-Regione Toscana, Pisa, Italy; 2 grid.8484.00000000417572064Clinica Pediatrica, Università di Ferrara, Ferrara, Italy; 3 grid.7644.10000000101203326Pediatric Unit, University of Bari, Bari, Italy; 4 grid.8158.40000000417571969Division of Hematology, Unit of Thalassemia, Policlinico Vittorio Emanuele, University of Catania, Catania, Italy; 5 Pediatria, Adolescentologia e Talassemia, Arcispedale "S.Anna", Ferrara, Italy; 6 Pediatric Hematology Unit, Policlinic Foundation San Matteo IRCCS, Pavia, Italy; 7 Servizio Radiologia Ospedaliera-Universitaria, Arcispedale "S. Anna", Ferrara, Italy

## Background

In 2004 seven Italian centers reported survival data for patients with thalassemia major (TM) and showed that heart disease due to iron overload was the most common cause of death (Borgna et al Haematologica 2004). In the same years the accurate and noninvasive assessment of cardiac siderosis was made possible in Italy by the introduction of the T2* cardiovascular magnetic resonance (CMR).

We aimed to evaluate if the deployment of T2* CMR had an impact on the mortality rate.

## Methods

Four centers contributed to the present study, updating the data of the enrolled patients until August 31, 2010. For the patients who died, the date of the death represented the end of the study. 577 patients (264 females and 313 males) were included.

## Results

One-hundred and fifty-nine (27.6%) patients died, 124 of whom (77.9%) died before the year 2000. MRI was not performed in 406 patients (70.4%) and no patient had been scanned before his/her death. Among the survivors, MRI was not performed in the 59% of the cases (P < 0.0001). The absence of an MRI scan was a significant univariate prognosticator for death (HR = 43.25, 95%CI = 11.32-165.33, P<0.0001).

**Figure 1:**
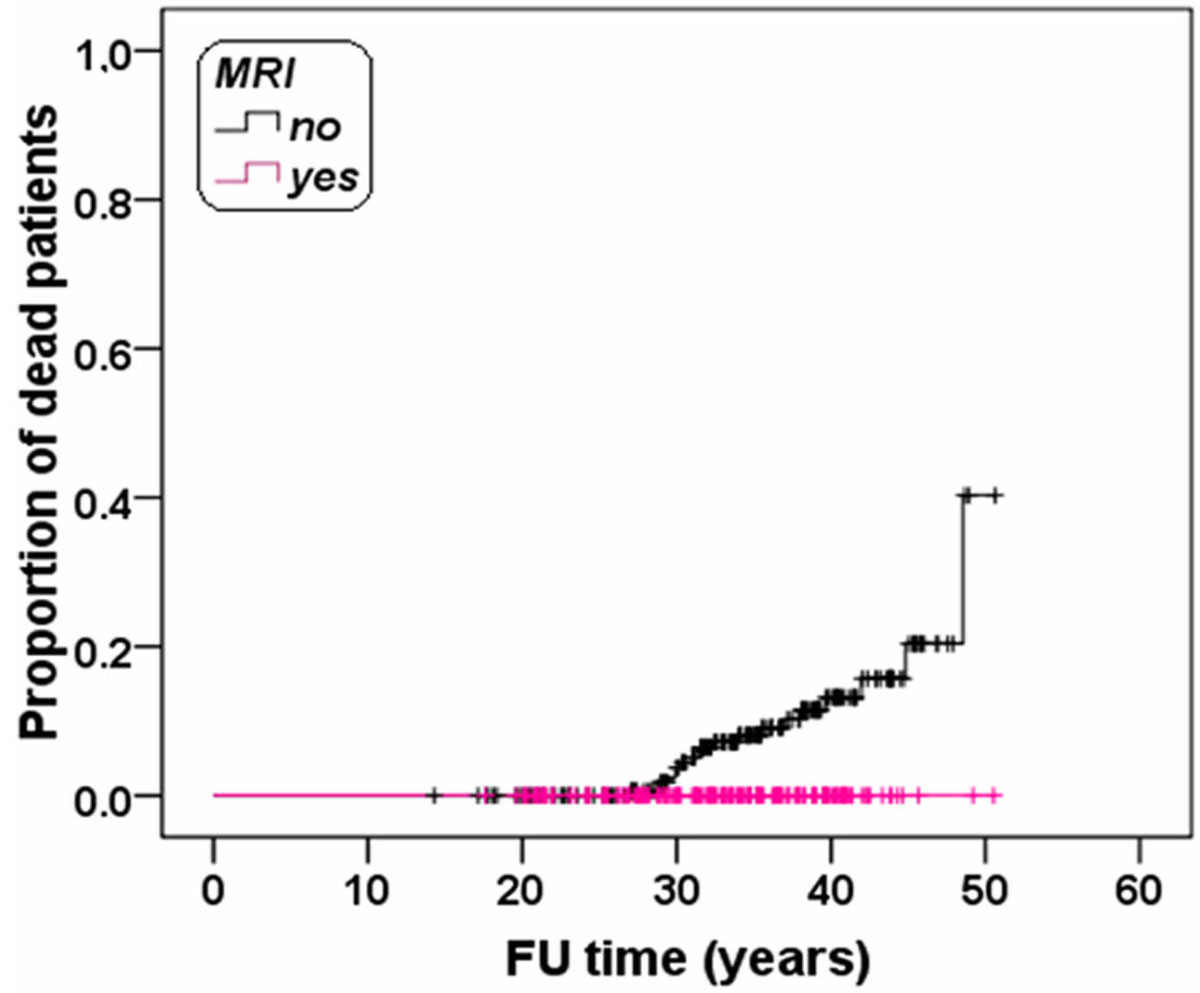
**Kaplan-Meier survival curve**.

The study was restricted to the patients dead after 2004 (19/159=12%) or followed until August 2010 (N = 357). In this subgroup of 376 patients, MRI was not performed in the 52.4% of the survivors and in all dead patients (P<0.0001). The absence of a MRI exam was reconfirmed as a strong predictive factor for death (HR=49.37, 95%CI = 1.08-2263.24, P = 0.046). The Kaplan-Meier curve is showed in Figure 1. The log-rank test revealed a significant difference in the curves (P < 0.0001).

## Conclusions

Our data suggests that the use of T2* CMR, that enables individually tailored chelation regimes reducing the likelihood of developing decompensated cardiac failure, allowed the reduction of cardiac mortality in chronically transfused TM patients.

